# Spatiotemporal Dynamics and Outbreak Risk of *Apolygus lucorum* in Semi-Arid Wine Grape Regions: An Analysis Based on Multi-Factor Drivers and Machine Learning Models

**DOI:** 10.3390/insects17070719

**Published:** 2026-07-11

**Authors:** Haiyan Chen, Jianying Zhang, Long Jia, Shenghu Su, Peiwen Gu, Xiaoyu Zhang

**Affiliations:** 1 School of Life Sciences, Ningxia University, Yinchuan 750021, China; nxdxchy@163.com (H.C.); zhjywa123@163.com (J.Z.); 2 School of Agriculture, Ningxia University, Yinchuan 750021, China; 13037955994@163.com (S.S.); gupeiwen2019@nxu.edu.cn (P.G.); 3 Institute of Meteorological Sciences, Ningxia Meteorological Bureau, Yinchuan 750002, China; zhang_xynet@163.com

**Keywords:** vineyard pest monitoring, LOWESS smoothing, phenological timing, adult trap catches, XGBoost-SHAP, partial dependence analysis

## Abstract

*Apolygus lucorum* is a major piercing–sucking pest in viticulture, yet its seasonal dynamics and outbreak risk in semi-arid wine-grape regions remain insufficiently understood. This study was conducted in a semi-arid wine-grape region of northwestern China during the 2024–2025 growing seasons. Adult density was monitored at 150 fixed sampling points across five landscape units. LOWESS-based phenological staging, stage-specific spatial interpolation, and an XGBoost-SHAP framework integrating meteorological, topographic, and grape phenological predictors were used to characterize spatiotemporal patterns and key predictors. *A. lucorum* density remained low in May, increased from June to July, peaked during August-September, and remained relatively high in October, with higher overall abundance in 2025 than in 2024. Spatial analyses revealed marked heterogeneity among landscape units, with high-density patches shifting across years and phenological phases. The XGBoost model showed good predictive performance, with an *R*^2^ of 0.878 on the independent test set and a mean GroupKFold cross-validation *R*^2^ of 0.869 ± 0.014. SHAP analysis identified grape phenology, elevation, relative humidity, sunshine duration, and temperature as the leading predictors of model-predicted density. PDP and ICE analyses showed higher predicted counts during later phenological periods, at lower elevations, and under higher relative humidity, particularly around 55%. Two-dimensional PDPs further indicated that high predicted densities mainly occurred under combinations of higher relative humidity, later phenological timing, moderate-to-high temperature, longer sunshine duration, and lower elevation. These findings provide a scientific basis for implementing precision-integrated pest management strategies in semi-arid viticultural regions, where monitoring relative humidity during critical phenological windows can serve as an early warning indicator for impending outbreaks.

## Simple Summary

This study examined the population dynamics of *Apolygus lucorum*, a pest insect that damages wine grapes, in a dry, semi-arid vineyard region. We followed its seasonal occurrence over two grape-growing seasons in a wine-grape production area of northwestern China, using pheromone traps placed at 150 fixed monitoring sites across five landscape units. The study showed that this pest was scarce in spring, increased gradually after early summer, and became most abundant from late summer to early autumn. However, the timing and location of high pest abundance differed among vineyard areas and between years, indicating that local landscape conditions can influence where outbreaks are most likely to develop. By combining field monitoring data with weather, terrain, and grapevine growth information, the model highlighted grapevine phenology, relative humidity, elevation, sunshine duration, and temperature as useful indicators for predicting adult pest abundance. In particular, humid conditions during later grapevine growth stages were associated with higher pest risk, while lower-elevation vineyard areas tended to show higher predicted abundance. These results suggest that growers should strengthen monitoring during humid periods in the middle to late growing season, rather than relying on fixed calendar dates alone. The approach developed here can support more timely pest surveillance and improve early-warning decisions in semi-arid wine-grape regions.

## 1. Introduction

Wine grapes are grown across the world’s golden viticultural belt. Their sustainable production and quality are critical to the wine industry, yet herbivorous insects remain a major problem in many regions [1,2]. Among these pests, the polyphagous *Apolygus lucorum* (Meyer-Dür, 1843) (Hemiptera: Miridae) poses a serious threat. This species overwinters as eggs within grape canes and weed stems, develops multiple overlapping generations per year, and its developmental rate is entirely temperature-dependent. Both nymphs and adults feed via piercing–sucking mouthparts on tender buds, young leaves and fruits, resulting in sustained damage to various crops including wine grapes, cotton, jujubes and apples [3,4,5,6,7]. During feeding, toxic saliva injected by *A. lucorum* triggers tissue perforation, leaf wilting, abscission of reproductive organs and fruit scarring [8,9], which directly impairs the photosynthetic capacity of host plants. Additional biological traits of this pest include its small body size, nocturnal activity, and seasonal host switching behavior [2]. Adult individuals exhibit long-distance migratory capacity, dispersing to suitable host vegetation for oviposition prior to overwintering [10,11]. Given the complex environmental gradients within agricultural landscapes, disentangling the drivers underlying spatio-temporal fluctuations of pest populations represents a core challenge for integrated pest management [12,13]. Conventional regression models fail to capture non-linear response thresholds and phenology-specific spatial heterogeneity of pest infestations, substantially limiting their utility for pest early-warning systems.

Previous studies have confirmed that meteorological conditions, topographic features, and host plant phenology profoundly influence insect population dynamics [14]. Climate factors directly affect insect survival, development, and reproduction [15,16]; topography shapes local microclimates and habitat conditions [12]; and phenological status determines resource availability and host selection preferences of pests [17]. Although the role of these environmental drivers is widely recognized, existing research falls into two main categories. Some studies analyze temporal dynamics alone while ignoring the spatial redistribution of pest populations [13,18]. Others produce static spatial distribution maps that fail to account for interannual phenological shifts [19]. Furthermore, very few analytical frameworks can simultaneously resolve multi-factor interactions, detect nonlinear thresholds, and quantify the contribution of each driver at different stages. Consequently, the mechanisms by which weather, topography, and phenology jointly shape the seasonal dynamics and spatial heterogeneity of *A. lucorum* at the landscape scale remain unclear, especially in semi-arid regions.

To address the above limitations, LOWESS smoothing is used to define pest phenological stages based on field population monitoring data rather than fixed calendar dates [20]. This data-driven division better reflects actual field conditions, as climate variability often causes shifts in outbreak timing across years [21]. Ordinary kriging is then applied to generate spatial distribution maps for different phenological stages, visually illustrating how high-risk areas evolve over the growing season [22]. This dynamic spatial analysis outperforms static distribution studies that fail to capture phenological movement. Finally, the combination of extreme gradient boosting and the SHAP model accurately identifies key environmental drivers, quantifies nonlinear response thresholds, and reveals interactive effects such as the synergy between humidity and host plant phenology [23,24,25]. Unlike traditional regression methods, this approach does not assume linearity or additivity and provides explicit threshold values for practical application [26]. Together, these three techniques form a coherent analytical pipeline that is both methodologically rigorous and readily transferable to other regions.

We selected a typical semi-arid montane alluvial fan system within the global viticultural golden latitude belt as a test platform. This experimental area is located in the wine grape region at the eastern foothills of the Helan Mountains in Ningxia, China. The foothills offer strong elevation, precipitation, and thermal gradients. This study aims to characterize the seasonal dynamics and spatial distribution of *A. lucorum* in a stage-specific manner, to quantify how spatial heterogeneity changes across different phenological windows, and to identify the key environmental drivers along with their precise thresholds and interactive effects using the XGBoost SHAP framework. By presenting this validated analytical pipeline, we provide a standardized and transferable methodology for establishing locally tuned early warning systems in other arid or semi-arid wine regions facing similar pest challenges.

## 2. Materials and Methods

### 2.1. Study Area Overview

The wine grape region on the eastern foothills of the Helan Mountains is located at 105°45′–106°47′ E, 37°43′–39°23′ N, in northern Ningxia, China [27]. It extends from Shizuishan City in the north to Hongsibu District in the south, bordered by the Yellow River to the east and flanked by the Helan Mountains to the west. The terrain gradually slopes downward from west to east, with the core cultivation area situated on an alluvial–proluvial plain between the Yellow River alluvial plain and the piedmont alluvial fans of the Helan Mountains. The area features predominantly flat topography with minimal relief, shallow and gently sloped gullies, and low soil erosion, providing optimal conditions for large-scale viticulture. The average elevation is over 1000 m above sea level [28,29]. The climate is characterized by dryness, cool temperatures, significant diurnal temperature variation, and abundant sunshine. The mean annual temperature is 9.8 °C, and annual precipitation ranges between 160 and 400 mm [30,31,32]. These conditions favor sugar accumulation and phenolic synthesis in grape berries. The primary soil type is light sierozem, supplemented by aeolian sandy soil and irrigated warp soil. Some plots contain a gravel component [10], with a predominant sandy loam texture [33]. This soil profile offers good permeability, featuring a porous sandy surface layer and a deep, well-structured yet friable subsoil, which effectively regulates water availability to the grapevines. These unique topographic and edaphic conditions collectively shape wine grape quality attributes and profoundly influence the habitat and reproductive environment of *A. lucorum*.

**Figure 1:**
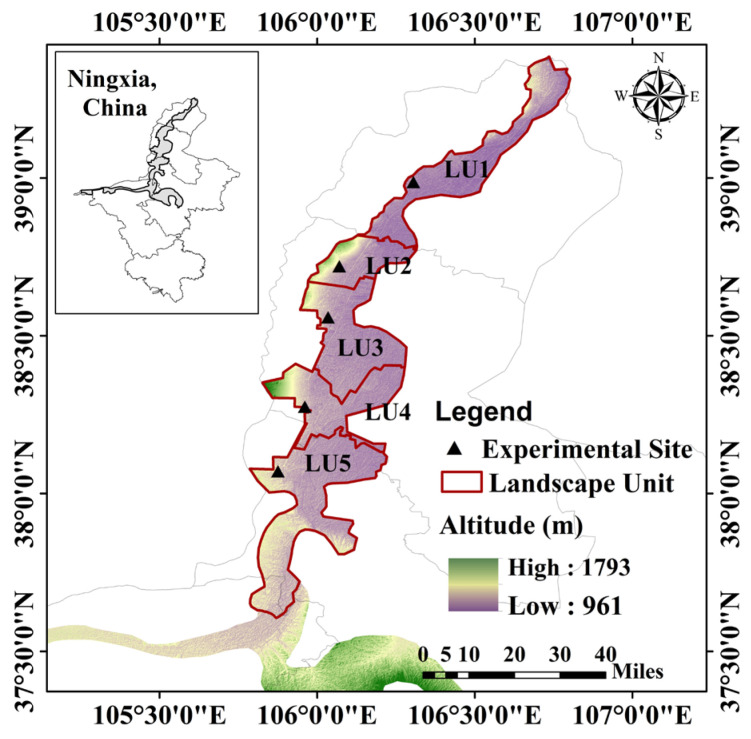
Location and distribution of landscape units (LU1–LU5) in the study area of the Ningxia Helan Mountain Eastern Foothill wine grape region, China. Black triangles represent experimental sites, and the color gradient indicates altitude. The inset shows the location of Ningxia within China.

To systematically investigate the population dynamics of *A. lucorum* under different spatial conditions, we established five landscape units (LUs) along a north–south transect covering the latitudinal range of the study area, designated from north to south as LU1 through LU5. Each landscape unit corresponds to a spatially well-defined large-scale vineyard sub-region, within which 30 fixed monitoring points were arranged in a regular grid with a minimum spacing of 50 m (Figure 1). These units capture the natural spatial variation in topography, soil, and microclimate across the eastern foothills of Helan Mountain, thereby facilitating the analysis of environmental drivers of *A. lucorum* occurrence across heterogeneous landscape systems.

### 2.2. Monitoring and Sampling of A. lucorum

Field monitoring of *A. lucorum* was conducted during two grape-growing seasons from 2024 to 2025 in five representative landscape units (LUs) in the eastern foothills of Helan Mountain. The five LUs were established along a north–south transect covering the full latitudinal range of the study area and were numbered from north to south as LU1 to LU5. Each LU represented a clearly defined large-scale wine-grape subregion. Within each LU, three representative vineyards were selected. In each vineyard, five quadrats were established using a five-point sampling method, and two monitoring points were set within each quadrat. Thus, 30 fixed monitoring points were established in each LU, resulting in a total of 150 fixed sampling points across the five LUs. Adjacent sampling points were separated by at least 50 m to reduce interference among traps, and one sex pheromone trap was deployed at each sampling point. Each trap was equipped with a species-specific sex pheromone lure for *A. lucorum*. The main active components of the lure were 4-oxo-(E)-2-hexenal and (E)-2-hexenyl butyrate, which were used to attract male adults of *A. lucorum*. The traps were hung within the grapevine canopy and maintained throughout the monitoring period. The pheromone lures and trapping devices were provided by Zhongjie Sifang Biotechnology Co., Ltd., Xi’an, China. Lures were replaced every 30 days according to the manufacturer’s instructions to ensure stable trapping efficiency. Field surveys were conducted every 10 days from early May to late October each year. Eighteen surveys were completed in each growing season, with the first survey conducted in early May (t_1_) and the last in late October (t_18_), resulting in a total of 36 surveys over the two years. During each survey, the number of male adults of *A. lucorum* captured in each trap was recorded, and the trapped insects were removed after counting. Population density was defined as the number of adults captured per trap per 10-day interval and expressed as adults·trap^−1^·(10 days)^−1^.

### 2.3. Environmental Data Collection

Meteorological data were obtained from the China Surface Climate Data Daily Dataset provided by the National Meteorological Science Data Center, China Meteorological Data Service Centre (https://data.cma.cn/, accessed on 10 March 2026). In this study, daily observations from meteorological stations across Ningxia were extracted for the period from 1 May to 31 October in 2024 and 2025. To obtain site-specific meteorological estimates for each sampling point, daily meteorological variables were spatially interpolated to the 150 sampling points using inverse distance weighting (IDW) based on station coordinates. For each interval between two successive *A. lucorum* surveys, the interpolated daily data were aggregated to calculate mean temperature (°C), mean relative humidity (%), mean wind speed (m/s), mean daily sunshine duration (h), and cumulative precipitation (mm) for each 10-day monitoring interval. The interpolated meteorological variables were then temporally matched with the *A. lucorum* population data from the corresponding sampling point and monitoring interval for subsequent model analysis. Topographic data were derived from the Advanced Spaceborne Thermal Emission and Reflection Radiometer Global Digital Elevation Model (ASTER GDEM, version 3), a 30 m spatial resolution digital elevation dataset downloaded from the Geospatial Data Cloud platform (https://www.gscloud.cn/, accessed on 4 April 2026). Key topographic variables, including elevation (m), slope (°), aspect, curvature, and topographic shadow index, were extracted for each sampling point using ArcMap version 10.8.1 (Esri Inc., Redlands, CA, USA). In this study, topographic factors were treated as terrain-related predictors and proxy variables for characterizing local spatial variation in environmental conditions across the study area. Grape phenology was monitored at the same locations as the *A. lucorum* sampling points. Within a 2 m radius of each trap, three wine grape vines with uniform growth, no obvious disease or pest symptoms, and moderate vigor were marked as permanent phenological observation plants to ensure representative assessments of vine growth within each sampling quadrat. Phenological stages were recorded using the Day of Year (DOY) format, where calendar dates were converted to sequential days (1 January = DOY 1, 31 December = DOY 365) [34,35,36]. This study recorded all phenological stages classified per standard specifications together with their corresponding Day of Year (DOY) values, as shown in Tables S1 and S2. All meteorological processing and phenological observations were synchronized with *A. lucorum* population surveys.

### 2.4. Data Analysis Methods

#### 2.4.1. Temporal Dynamics and Key Occurrence Period Fitting of *A. lucorum* Populations

The survey period was grouped by month, and the median and interquartile range (IQR) of *A. lucorum* population density were calculated using Python version 3.13.7 (Python Software Foundation, Wilmington, DE, USA), Pandas and NumPy to characterize its monthly distribution pattern [37,38]. Population temporal dynamics were smoothed using Locally Weighted Scatterplot Smoothing (LOWESS) implemented in Statsmodels, with a smoothing fraction of 0.4 and three robustness iterations. Based on the LOWESS-smoothed curve, three key occurrence periods were defined as Phase I, the rapid growth phase, from the date when the smoothed density first consistently exceeded 10% of the seasonal peak until it reached 90% of the peak, Phase II, the peak phase, during which the smoothed density remained above 90% of the peak, and Phase III, the decline phase, from the date when the density dropped below 90% of the peak until the end of the season.

A systematic sensitivity analysis was implemented to quantify how the selected smoothing fraction and percentage thresholds altered the delineation of key phenological phases. Four levels of smoothing fraction (0.25, 0.30, 0.40 and 0.50) were combined with three threshold pairs for phase partitioning: (10%, 90%), (20%, 80%) and (25%, 75%), generating a total of 12 distinct parameter sets. For each parameter set, we calculated the onset, peak and decline timings across all wineries in both study years. The minimum, maximum, range and standard deviation of each critical timing metric were computed to measure the magnitude of bias induced by parameter perturbation. Narrow ranges and low standard deviations across all configurations indicated low parameter sensitivity, verifying the reliability of phase segmentation derived from the baseline parameters (frac = 0.4, 10%/90% thresholds).

#### 2.4.2. Analysis of Spatial Distribution Patterns

Ordinary kriging interpolation was performed to generate continuous population density surfaces for different survey years, landscape units (LUs), and phenological phases. For each combination of survey year, LU, and phenological phase, experimental variograms were first calculated using field-measured density data from sampling points. Three commonly used theoretical models, namely spherical, exponential, and Gaussian models, were then fitted to the experimental variograms. Model parameters were estimated using the weighted least squares method, and the optimal variogram model for each combination was selected based on the minimum weighted residual sum of squares (WRSS). The selected model type and its corresponding nugget, partial sill, total sill, range, and WRSS values were recorded for each combination.

Leave-one-out cross-validation (LOOCV) was implemented to evaluate the predictive accuracy of the kriging models. In this procedure, a single sampling point was iteratively withheld from the dataset, and a new kriging model was calibrated using the remaining observations to predict the density value at the excluded location. Predicted densities were subsequently compared with field measurements to quantify model performance. The validation metrics included the mean error (ME), mean absolute error (MAE), root mean square error (RMSE), root mean square standardized error (RMSSE), and coefficient of determination (*R*^2^). Specifically, ME reflected overall prediction bias; MAE and RMSE indicated the magnitude of prediction errors; RMSSE assessed the consistency between model-derived standard errors and empirical prediction errors; and *R*^2^ represented the explanatory power of predicted values for the spatial variation in observed population densities.

Global Moran’s I statistic was applied to quantitatively detect spatial autocorrelation for each Year-LU-Phase combination. A k-nearest neighbor spatial weight matrix was built based on sampling coordinates, and statistical significance was calculated through 999 permutation tests. A significance threshold of *p* ≤ 0.05 was set to identify obvious spatial autocorrelation. Groups with significant Moran’s I values exhibited distinct spatial clustering patterns, whose spatial distribution characteristics were further interpreted in combination with kriging interpolation outputs. In contrast, non-significant Moran’s I results indicated no detectable global spatial autocorrelation, and bubble charts of field-measured point densities were used as an alternative. Maps derived from such non-significant groups were excluded from the discussion of hot spot shifts. All kriging interpolation procedures and the generation of population density distribution maps were implemented in ArcMap version 10.8.1 (Esri Inc., Redlands, CA, USA) with the Geostatistical Analyst extension.

#### 2.4.3. XGBoost-SHAP Modeling and Interpretation of *A. lucorum* Population Density

To identify environmental predictors associated with the seasonal dynamics and spatial distribution of *A. lucorum*, an XGBoost regression model was constructed using spatiotemporal monitoring data from 150 fixed sampling points across five landscape units (LUs). The response variable was population density at the 10-day monitoring scale. For each sampling point and monitoring interval, population density was paired with meteorological variables from the same period, topographic variables extracted at the sampling location, and phenological information represented by day of year (DOY). A total of 11 predictors were included, covering meteorological, topographic, and phenological variables [12,39]. Data processing and integration were performed using Python version 3.13.7 (Python Software Foundation, Wilmington, DE, USA), pandas and numpy, resulting in a final dataset of 5400 observations.

Before model training, variance inflation factors (VIFs) were calculated for the 11 predictors to assess potential multicollinearity. VIFs were computed from the standardized predictor matrix, and VIF > 10 was used as the threshold for severe multicollinearity.

To reduce the risk of data leakage caused by repeated observations at the same location, sampling point was used as the grouping variable when splitting the dataset into training and test sets at a 7:3 ratio. This ensured that all observations from the same fixed sampling point were assigned exclusively to either the training or test set. The resulting training and test sets contained approximately 3780 and 1620 observations, respectively. The XGBoost regression model was implemented using the xgboost library with the squared-error regression objective (objective = “reg:squarederror”). Model parameters were set as follows: n_estimators = 100, max_depth = 4, learning_rate = 0.05, subsample = 0.7, colsample_bytree = 0.7, reg_alpha = 0.5, and reg_lambda = 0.5. The random seed was fixed at 42, and all other parameters were kept at their default values. Model stability was evaluated using five-fold GroupKFold cross-validation with sampling point ID as the grouping variable [40]. Model performance was evaluated using the coefficient of determination (*R*^2^), root mean square error (RMSE), and mean absolute error (MAE), all computed with scikit-learn. SHAP values were calculated from the trained model to interpret the contribution of each predictor to model predictions. Model persistence was performed using joblib [41,42].

To elucidate the contribution of individual features to model predictions, the SHapley Additive exPlanations (SHAP) method was applied [43]. SHAP values were calculated for each sample using the shap library to quantify feature contributions from both global and local perspectives [44]. Features were ranked according to their mean absolute SHAP values, computed with numpy. To visualize model response patterns of key predictors, partial dependence plots (PDPs) and individual conditional expectation (ICE) plots were generated using the sklearn.inspection module [45]. Based on SHAP importance rankings, the six most influential features were selected for PDP and ICE analysis. For each feature, 50 uniformly spaced grid points were defined within the 5th to 95th percentile range of its observed values using numpy. PDPs were used to summarize the average marginal model response, whereas ICE curves were used to display sample-level response heterogeneity [46].

To quantify uncertainty in PDP curves, cluster bootstrap resampling was performed at the sampling-point level. Specifically, sampling points were resampled with replacement 500 times, PDP curves were recalculated for each bootstrap replicate, and the 2.5th and 97.5th percentiles of the bootstrapped values were used to construct 95% uncertainty bands. To visualize joint response patterns among influential predictors, two-dimensional partial dependence plots (2D PDPs) were generated using the sklearn.inspection module [45]. Predictor pairs were selected based on SHAP importance rankings. For each pair, the joint response surface was calculated within the 5th to 95th percentile ranges of the observed values of both predictors. The 2D PDPs were used to visualize the joint marginal model response of selected predictor pairs. To quantify pairwise interaction strength beyond visual interpretation, SHAP interaction values were computed using the shap.TreeExplainer method. For each observation, the SHAP interaction value matrix decomposed the contribution of each feature into its main effect and its interaction with every other feature. The mean absolute SHAP interaction value was then calculated for all feature pairs across the full dataset, and the top 20 pairs with the highest values were reported to identify the most influential predictor interactions.

## 3. Results

### 3.1. Temporal Dynamics and Fitted Model of A. lucorum Populations

**Figure 2:**
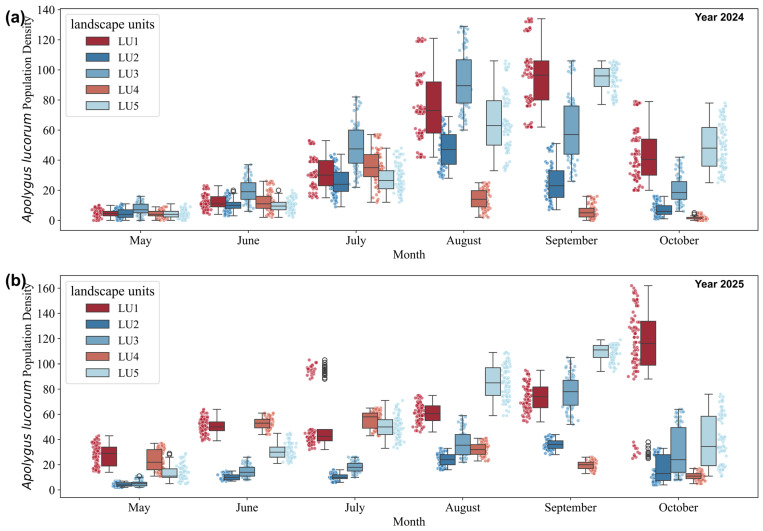
Seasonal and interannual dynamics of *A. lucorum* population density across five landscape units during 2024–2025. (**a**) Population density in 2024; (**b**) population density in 2025. Box plots show the median, interquartile range, and range of *A. lucorum* counts per sampling unit, with overlaid scatter points representing individual observations. LU1–LU5 denote the five distinct landscape units. Data are expressed as adults per trap per 10-day interval (adults·trap^−1^·(10 d)^−1^).

The population density of *A. lucorum* exhibited a stable seasonal variation pattern during 2024–2025 (Figure 2). Population abundance remained low in May, increased steadily from June to July, and peaked sharply from August to September, followed by a slight decline but sustained high density in October. Significant interannual variations were detected, with overall population density distinctly higher in 2025 relative to 2024, particularly in the late growing season spanning August to October (Figure 2b).

Marked spatial heterogeneity in pest density was observed among the five landscape units across all sampling periods. LU1 possessed the highest annual population density, with the peak value recorded in October 2025. LU5 showed the second-highest density, whereas LU3 and LU4 maintained moderate population levels. LU2 consistently presented the lowest monthly density, accompanied by lower median and interquartile range values than other units. Box plot analysis demonstrated substantially expanded interquartile ranges and abundant high outliers during the August-October outbreak stage, suggesting intense population fluctuations and localized pest outbreaks. Density data displayed a right-skewed distribution in most months. The natural population of *A. lucorum* was generally maintained at low and moderate levels, and extremely high population density merely emerged during seasonal outbreak periods.

**Figure 3:**
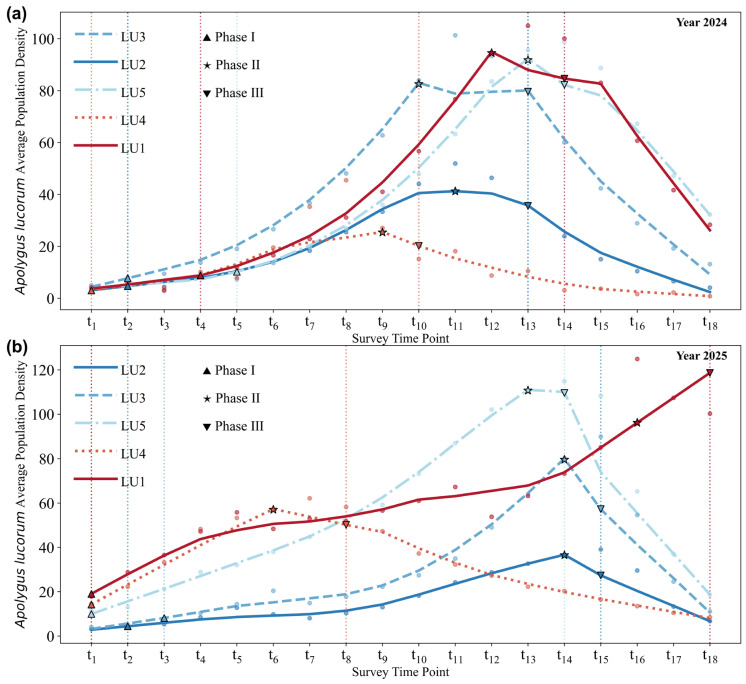
LOWESS-smoothed curves of the average population density of *A. lucorum* at 18 survey time points (t_1_–t_18_) for each landscape unit (LU1–LU5) in 2024 (**a**) and 2025 (**b**). Scatter points represent individual raw observations. Based on the smoothed curves and relative to the seasonal peak density, three key occurrence periods were defined: Phase I (rapid growth phase), Phase II (peak phase), and Phase III (decline phase).

The LOWESS-smoothed curves revealed distinct temporal dynamics of *A. lucorum* among landscape units and between years (Figure 3). In 2024, most landscape units showed a relatively clear increase–peak–decline pattern. Population density increased during the early survey period, reached a high-abundance stage in the middle to late season, and then declined toward the end of the season. LU1 and LU5 exhibited relatively high peak densities, whereas LU2 maintained a lower population level throughout the season. LU3 showed an intermediate pattern, while LU4 had an earlier and shorter period of population increase followed by a rapid decline. In 2025, the population trajectories were more asynchronous among landscape units. LU4 reached its peak relatively early and then declined continuously, whereas LU3 and LU5 showed later increases and entered the high-abundance period in the middle to late season. LU1 displayed a prolonged increasing trend and reached its highest density near the end of the survey period, suggesting delayed population development. These results indicate that the timing of rapid growth, peak abundance and decline varied substantially among landscape units and between years.

The sensitivity analysis indicated that phase delineation was generally robust to changes in LOWESS smoothing fraction and threshold combinations, but the stability differed among phase boundaries (Tables S3 and S4). Peak timing was the least sensitive to parameter perturbation, with relatively narrow ranges and low standard deviations across most landscape units. In contrast, the onset of Phase I showed greater variability, particularly in 2025 LU3 and 2025 LU2, where the early-season increase was more gradual or asynchronous. Decline timing showed intermediate sensitivity. Overall, the baseline parameter setting, frac = 0.4 with 10%/90% thresholds, provided a reliable basis for defining the three occurrence phases used in subsequent spatial analyses.

### 3.2. Spatial Distribution and Hotspot Variation of A. lucorum Populations

**Figure 4:**
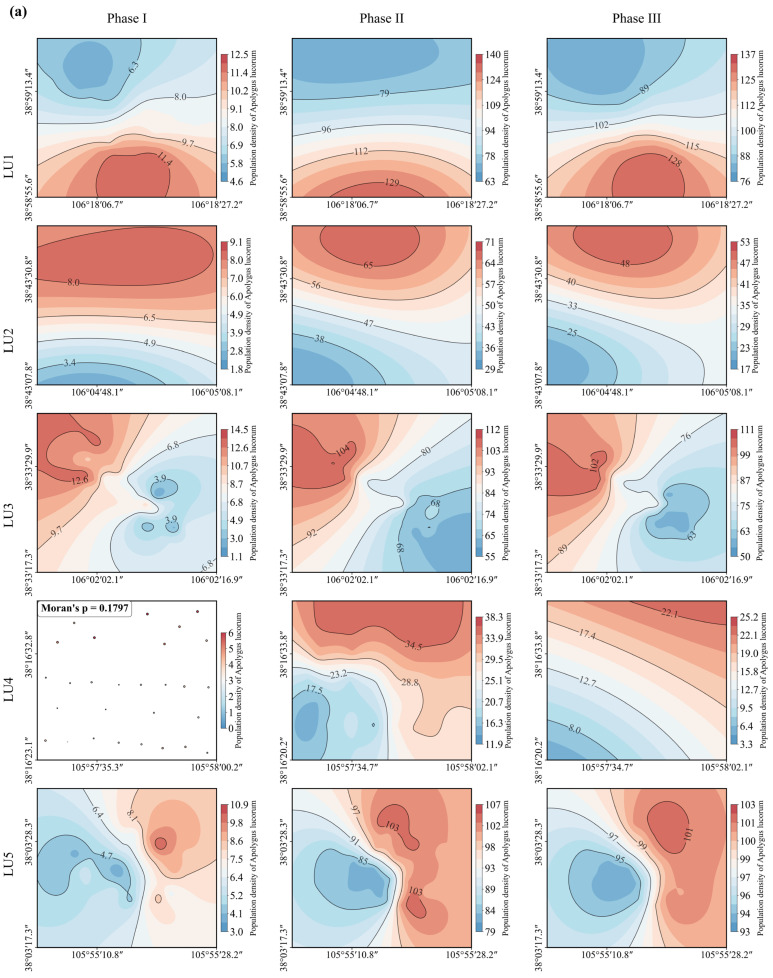

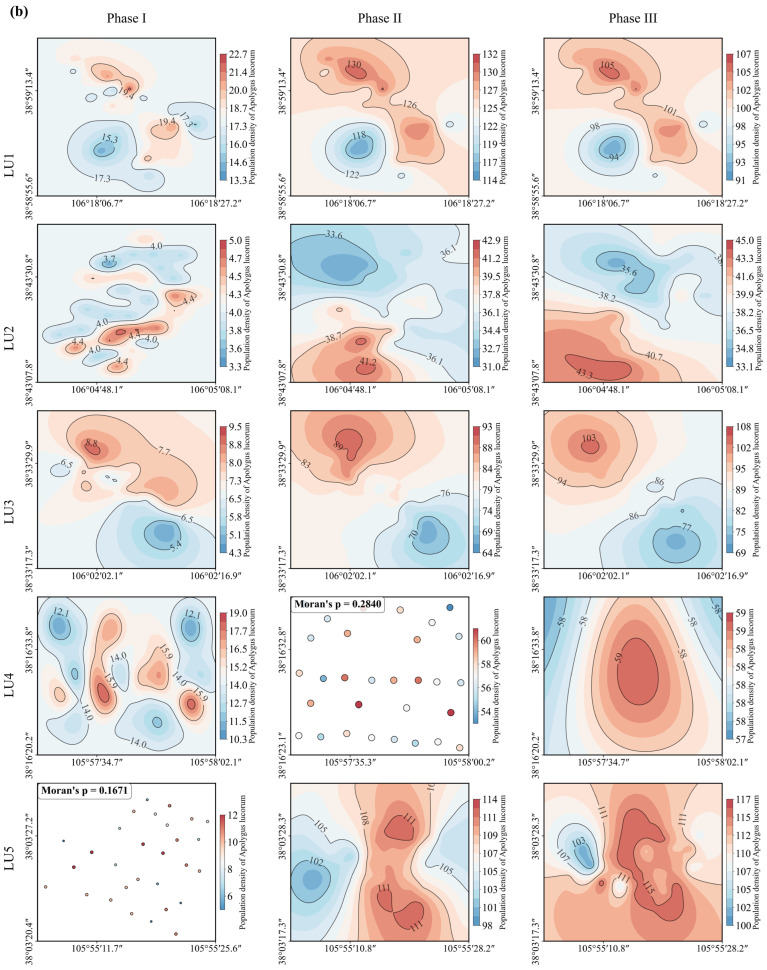
Spatial distribution contour plots of *A. lucorum* population density across landscape units (LU1–LU5) during three occurrence phases in 2024 (**a**) and 2025 (**b**). Phase I, Phase II, and Phase III correspond to the rapid growth, peak, and decline phases, respectively. Filled colors and contour lines represent interpolated population density gradients, and labeled values indicate observed densities at monitoring points. Color bars denote the population density scale for each panel. Panels without significant Moran’s I spatial autocorrelation are shown as observed point-density maps only and were excluded from hotspot interpretation.

Significant spatial heterogeneity in the population density of *A. lucorum* across landscape units (LUs) and phenological phases is collectively evidenced in (Figure 4a,b), where the geographic positions of high-density patches and their interphase shifts display marked LU-specific traits. For the 2024 dataset shown in (Figure 4a), LU1 formed a clear spatial gradient with high densities in the southern portion and low densities in the north; high-density aggregations of LU2 were mainly confined to northern and northeastern zones; LU3 recorded peak density values across western and northwestern areas; dense patches extended from the north to northeast of LU2 throughout Phase II and Phase III, while high-density clusters of LU5 predominantly occupied eastern and northeastern regions.

Spatial configurations of individual LUs were not fully consistent when contrasted against the patterns presented in Figure 4b. High-density hotspots of LU1 remained stably distributed in northern and northeastern extents across all three phenological stages, and LU3 sustained a fixed spatial framework featuring high densities in the north-northwest and low densities in the southeast, indicating temporally stable dense population patches within these two LUs. In contrast, high-density zones of LU2 migrated toward southern and southwestern areas during Phase II and Phase III, reflecting distinct phase-dependent spatial variation. LU4 exhibited discrete patchy density distributions in Phase I, with an obvious central high-density core emerging in Phase III, whereas dense assemblages in LU5 clustered within central and eastern territories in Phase II and Phase III.

Comparative analysis of the two spatial datasets illustrated in Figure 4a,b reveals that the locations of *A. lucorum* spatial aggregations are jointly regulated by landscape unit, survey year and phenological phase. Within a single LU, high-density domains may either retain static geographic positions across the growing season or experience distinct spatial translocation between successive phases. Critically, not all phenological phases can serve as valid evidence for hotspot distribution or interphase hotspot migration. The global Moran’s I statistic returned non-significant results for LU4 Phase I in Figure 4a (*p* = 0.1797), and no detectable significant spatial autocorrelation was observed for LU4 Phase II and LU5 Phase I in (Figure 4b). These non-significant combinations were only reserved for descriptive visualization of field-measured point densities and excluded from all hotspot interpretations derived from kriging interpolation.

Variations in the optimal semivariogram models fitted to *A. lucorum* population density across different survey years, landscape units, and phenological phases indicate that the spatial correlation structure of *A. lucorum* density was not constant, but varied among years, landscape units, and phenological phases (Table S5). The leave-one-out cross-validation results were used to evaluate the predictive reliability of the ordinary kriging models (Table S6). Spatial autocorrelation and hotspot-related statistics were used to identify combinations suitable for hotspot interpretation (Table S7).

Statistically significant Moran’s I values were obtained for most combinations of landscape unit and phenological phase, suggesting that *A. lucorum* population density generally showed an aggregated rather than random spatial pattern. For the 2024 sampling year, most groups exhibited significant spatial autocorrelation, with the exception of LU4 Phase I. LU1, LU2, LU3, and LU5 showed relatively stable spatial aggregation or hotspot-related characteristics across multiple phases, supporting the interpretation of high-density zones shown in the corresponding kriging maps. In 2025, several phases of LU2, LU3, and LU4 also showed interpretable spatial patterns when both Moran’s I significance and kriging validation performance were considered. In particular, LU2 Phase III, LU3 Phase III, LU4 Phase I, and LU4 Phase III provided reliable interpolation outputs for spatial interpretation. In addition, LU2 Phase II showed a relatively high hotspot proportion, suggesting a broader distribution of high-density patches during this period.

Nevertheless, non-significant Moran’s I values were detected for LU4 Phase I in 2024, LU4 Phase II in 2025, and LU5 Phase I in 2025. These combinations were therefore used only for descriptive visualization of field-measured point density data and were excluded from interpretations of significant spatial hotspots and hotspot-shift patterns. Collectively, the variogram fitting results, cross-validation metrics, and spatial autocorrelation and hotspot statistics provide the statistical basis for interpreting the two-year spatial distribution maps. These results indicate that *A. lucorum* populations exhibited spatial aggregation in most landscape unit and phenological phase combinations, whereas aggregation intensity, interpolation reliability, and hotspot proportion varied among survey years, landscape units, and phenological phases.

### 3.3. Identifying Environmental Drivers Using XGBoost-SHAP

#### 3.3.1. XGBoost Model Construction

Before constructing the XGBoost model, multicollinearity among meteorological, phenological, and topographic predictors was evaluated using the variance inflation factor (VIF). All predictors showed VIF values below the commonly used threshold of 5, indicating that severe multicollinearity was not present among the selected variables. Among the topographic variables, VIF values were low, ranging from 1.095 for curvature to 1.412 for aspect. Meteorological variables also showed acceptable VIF values, with relative humidity having the highest value within this category (VIF = 3.434), followed by temperature (VIF = 1.547), sunshine duration (VIF = 1.426), wind speed (VIF = 1.265), and precipitation (VIF = 1.158). The phenological predictor, grape phenological DOY, had the highest VIF among all variables (VIF = 3.956), but it remained below the threshold indicating problematic collinearity. Therefore, all selected predictors were retained for subsequent XGBoost modelling (Table 1).

**Figure 5:**
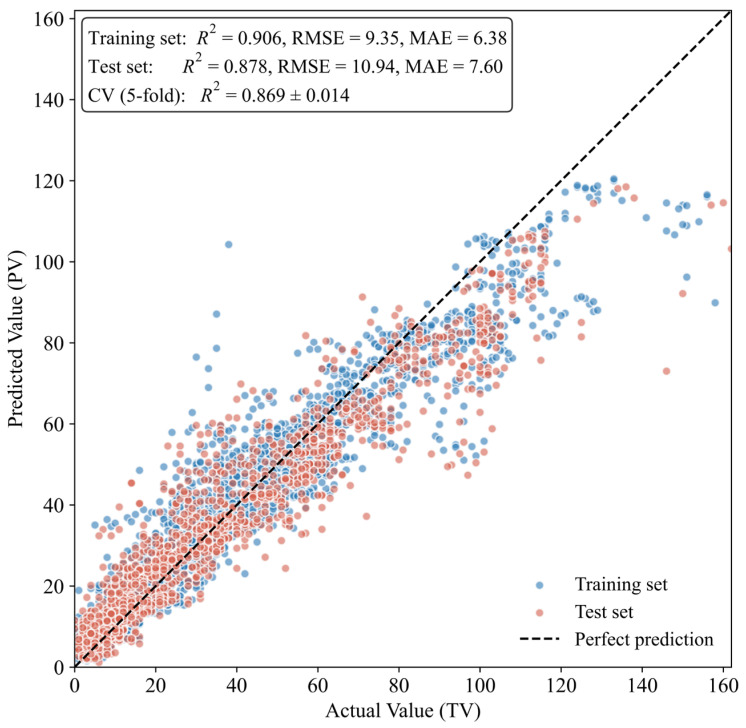
Observed vs. predicted population density of *A.lucorum* from the XGBoost model. Scatter points represent the training set (blue) and test set (red), respectively. The dashed line indicates the 1:1 perfect prediction. Model performance metrics include training set (*R*^2^, RMSE, MAE), test set (*R*^2^, RMSE, MAE), and 5-fold CV (*R*^2^).

The XGBoost regression model exhibited favorable predictive performance for the population density of *A. lucorum*. The predicted values were generally distributed close to the 1:1 reference line, indicating good agreement between observed and predicted population densities. For the training set, the model achieved an *R*^2^ of 0.906, with an RMSE of 9.35 and an MAE of 6.38. For the independent test set, the model retained a high predictive accuracy, with an *R*^2^ of 0.878, an RMSE of 10.94, and an MAE of 7.60. The five-fold GroupKFold cross-validation produced an average *R*^2^ of 0.869 ± 0.014, suggesting stable model performance across validation folds. The relatively small difference between the training and test performance indicates that the model did not exhibit strong overfitting. Overall, these results indicate that the XGBoost model captured the major spatiotemporal variation in *A. lucorum* population density and provided a reliable basis for subsequent SHAP-based interpretation of environmental predictor contributions (Figure 5).

#### 3.3.2. SHAP-Based Predictor Contributions to XGBoost Predictions of *A. lucorum* Population Density

**Figure 6:**
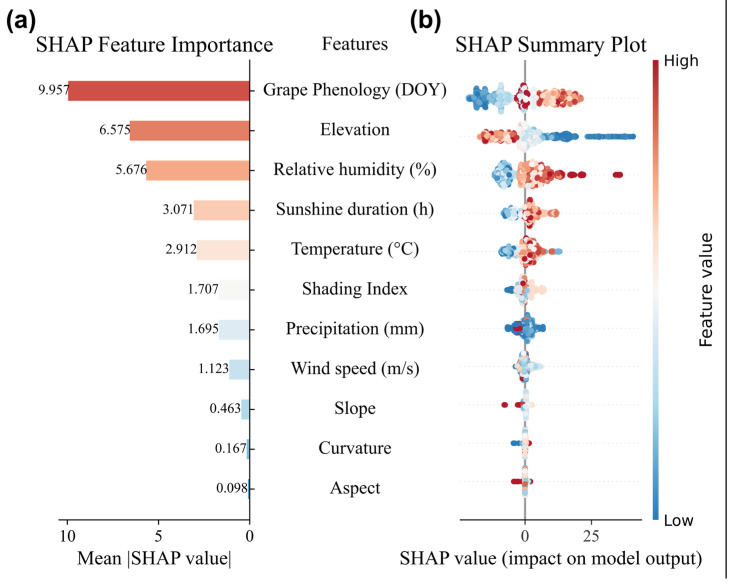
SHAP-based interpretation of predictor contributions to the XGBoost model. (**a**) Mean absolute SHAP values showing the relative contribution of each predictor to model output. (**b**) SHAP summary plot showing the direction and distribution of predictor contributions. Each point represents one observation, and colors indicate predictor values from low blue to high red. Positive SHAP values indicate increased model-predicted population density, whereas negative values indicate decreased predictions. DOY, day of year.

The SHAP feature-importance analysis showed that grape phenology, represented by day of year (DOY), contributed most strongly to model predictions, with the highest mean absolute SHAP value (9.957). Elevation ranked second (6.575), followed by relative humidity (5.676), sunshine duration (3.071), and temperature (2.912). The remaining predictors, including shading index, precipitation, wind speed, slope, terrain curvature, and aspect, exhibited relatively low contributions to model output (Figure 6a). The SHAP summary plot further indicated the direction and distribution of predictor contributions. Higher values of DOY, relative humidity, and sunshine duration were generally associated with positive SHAP values, corresponding to increased model-predicted population density of *A. lucorum*. In contrast, higher elevation values were mainly associated with negative SHAP values. Temperature displayed a more heterogeneous pattern, with SHAP values distributed on both sides of zero (Figure 6b).

#### 3.3.3. Partial Dependence and ICE Analysis of Key Predictors for *A. lucorum* Population Density

**Figure 7:**
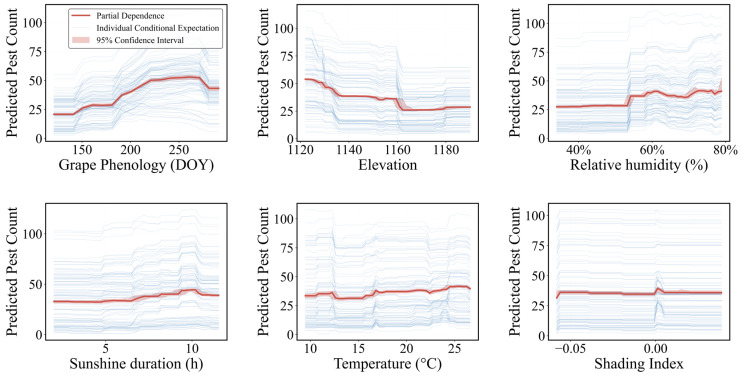
Partial dependence and individual conditional expectation plots for key predictors in the XGBoost model. Red lines indicate partial dependence plots (PDPs), light blue lines indicate individual conditional expectation (ICE) curves, and shaded areas indicate 95% confidence intervals. The plots show model-predicted pest count responses to grape phenology (DOY), elevation, relative humidity, sunshine duration, temperature, and shading index. DOY, day of year.

Grape phenology represented by DOY showed a clear increasing response pattern. The partial dependence curve remained relatively low at early phenological stages and increased markedly after approximately DOY 190–200, with higher model-predicted pest counts observed during later phenological periods. Elevation exhibited an overall negative response pattern: the predicted pest count decreased as elevation increased, with a sharp decline occurring at approximately 1160 m, and predicted pest abundance remained relatively low at higher elevations. Relative humidity showed a positive response pattern, with model-predicted pest count increasing around the 55% humidity range and remaining relatively higher under more humid conditions. Compared with DOY, elevation, and relative humidity, sunshine duration and temperature showed more moderate response patterns. Predicted pest count increased slightly with longer sunshine duration and higher temperature, but the overall changes were less pronounced. The shading index showed a nearly flat partial dependence curve, suggesting a limited contribution to variation in model predictions within the observed range. The ICE curves indicated substantial sample-level heterogeneity, particularly for DOY, elevation, relative humidity, and sunshine duration (Figure 7).

#### 3.3.4. Interactive Environmental Effects on *A. lucorum* Population Density

**Figure 8:**
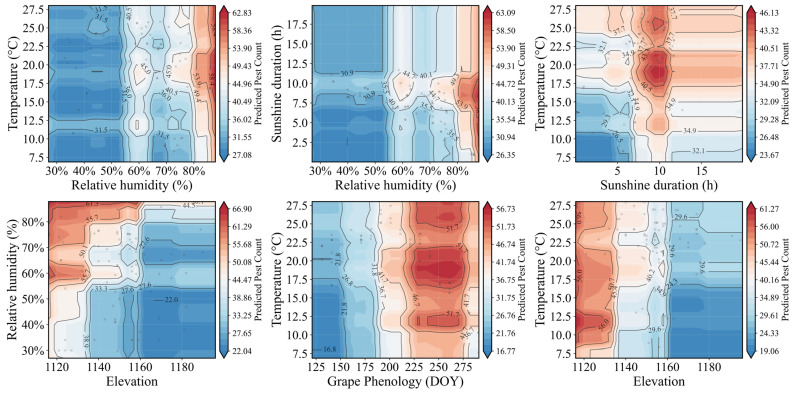
Two-dimensional partial dependence response surfaces of model-predicted pest count for pairwise combinations of temperature and relative humidity, sunshine duration and relative humidity, temperature and sunshine duration, relative humidity and elevation, temperature and grape phenology (DOY), and temperature and elevation. The color gradient from blue to red represents increasing model-predicted pest count, contour lines indicate equal prediction levels, and points show the distribution of samples in the corresponding variable space. Higher predicted values mainly occurred under combinations of higher relative humidity, higher temperature, longer sunshine duration, later grape phenological timing, and lower elevation. DOY, day of year.

In the relative humidity–temperature response surface, the model-predicted pest count generally increased with increasing humidity and reached higher levels under combinations of high humidity and relatively high temperature. The relative humidity–sunshine duration response surface showed that higher predicted pest counts occurred when high relative humidity coincided with longer sunshine duration. For the relative humidity–elevation pair, higher predicted values were mainly associated with higher humidity and lower elevation, whereas higher elevations corresponded to relatively lower predicted values. The temperature–DOY response surface showed that later phenological periods were associated with higher model-predicted pest counts, and this pattern was more evident under moderate to high temperature conditions. For the temperature–elevation pair, lower elevations generally corresponded to higher predicted values, whereas higher elevations were associated with lower predicted values. In contrast, the temperature–sunshine duration response surface showed a more complex pattern, with higher predicted values concentrated within specific combinations of temperature and sunshine duration. Overall, higher predicted values mainly occurred under combinations of higher relative humidity, higher temperature, longer sunshine duration, later phenological timing, and lower elevation (Figure 8). The corresponding SHAP interaction values for the main feature pairs are provided in Supplementary Table S8.

## 4. Discussion

### 4.1. Spatio-Temporal Heterogeneity of A. lucorum Populations

The spatio-temporal variation in *A. lucorum* populations observed in this study suggests that population occurrence in the semi-arid wine-grape region is likely influenced by the combined effects of regional climatic conditions, grape phenology, and local habitat heterogeneity within landscape units [47]. Temporally, *A. lucorum* showed a gradual increase in population density followed by a relatively delayed peak, which differed from the occurrence patterns reported in more humid grape-growing areas [48,49]. This difference may be related to the seasonal coordination between climatic suitability and host plant development [50,51,52]. In the early growing season, relatively low temperatures and limited precipitation may restrict the availability of suitable microhabitats and host plant resources during grape bud break and leaf expansion, thereby contributing to the low initial density of *A. lucorum* [53]. In contrast, during mid-to-late summer, more favorable thermal conditions and increased humidity associated with periodic precipitation coincided with the berry expansion-to-ripening period of grapevines [50,51,52]. This period may provide more suitable feeding and reproductive conditions for *A. lucorum*, which could partly explain the delayed population peak observed in this region.

Spatially, the localized aggregation and heterogeneous distribution of *A. lucorum* among landscape units indicate that population distribution was not spatially uniform. These patterns may be associated with differences in habitat suitability, host plant growth status, and local microenvironmental conditions [47,54]. Although the generally flat terrain of the study area may facilitate pest dispersal, variation in temperature, humidity, vegetation condition, and host phenological stage among sampling areas may create spatially heterogeneous suitability patches. Wind conditions may also influence the local spread of adult *A. lucorum* by facilitating or constraining short-distance movement among adjacent host patches, depending on wind speed and direction [55,56]. Areas with relatively favorable microclimatic conditions and host resources are likely to support higher local densities of *A. lucorum* [47]. The spatial redistribution of *A. lucorum* across phenological phases may also be associated with changes in host plant quality during grape development. As grape tissues mature and the availability of tender tissues changes, *A. lucorum* may shift toward areas where host conditions remain more favorable. However, because individual movement was not directly tracked in this study, this interpretation should be regarded as a plausible ecological explanation rather than direct evidence of dispersal behavior. Differences among landscape units may therefore reflect the combined influence of local habitat heterogeneity and temporal changes in host suitability. More heterogeneous habitats may provide multiple suitable patches and result in more complex spatial distribution patterns [54], whereas relatively homogeneous habitats may maintain more stable aggregation zones [57].

### 4.2. Environmental Factors Associated with A. lucorum Population Dynamics and Their Interactions

Grape phenology was identified as an important factor associated with *A. lucorum* population dynamics. Its influence is likely related to temporal changes in host resource availability. The population dynamics of herbivorous insects are often synchronized with host plant developmental stages, particularly changes in tissue tenderness, nutritional quality, and the availability of feeding sites [17,58]. Wine grapes at different growth stages may differ in nutrient composition and the abundance of tender tissues, which can affect host selection, feeding preference, and reproductive performance of *A. lucorum* [59,60]. In this study, the effect of phenology appeared to be closely linked with humidity conditions, suggesting that host suitability may be expressed more strongly when moisture conditions are favorable. Adequate moisture can promote normal grapevine growth and help maintain suitable host conditions, thereby indirectly supporting population growth of *A. lucorum* [61,62].

Relative humidity was also an important environmental factor associated with population variation. In arid and semi-arid regions, moisture availability is often a key constraint on the survival, development, and reproduction of piercing–sucking insects [63,64]. The results of this study suggest that *A. lucorum* density may respond nonlinearly to humidity conditions. Population density remained relatively low when relative humidity was below approximately 55%, whereas higher densities tended to occur when humidity exceeded this level. This pattern indicates that increased humidity may alleviate moisture limitation and improve habitat suitability for *A. lucorum*. Nevertheless, the threshold value should be interpreted as a model-derived estimate rather than a fixed physiological boundary.

Elevation was another important variable associated with the spatial distribution of *A. lucorum*. Elevation can influence local temperature, humidity, wind exposure, and vegetation growth conditions, thereby indirectly affecting insect habitat suitability [65,66]. In this study, population density tended to be higher in lower-elevation areas and declined when elevation exceeded approximately 1160 m. This pattern suggests that lower-elevation areas may provide more favorable microenvironmental conditions for *A. lucorum* occurrence. However, elevation itself is unlikely to act independently; rather, it probably represents the combined effects of several correlated environmental factors.

Temperature, precipitation, and sunshine hours had relatively smaller individual contributions, but they may still regulate population dynamics through indirect or interactive effects. Temperature determines the general range of insect development and activity [67], precipitation can modify field humidity and host plant water status [68], and sunshine duration may influence both microclimatic conditions and grapevine growth [69]. These factors may not act independently but instead interact with phenology, humidity, and elevation to shape the spatio-temporal distribution of *A. lucorum*.

Overall, the results indicate that *A. lucorum* population dynamics in this semi-arid wine-grape region are likely regulated by a combination of relatively stable environmental gradients and seasonally dynamic factors. Variables such as elevation and temperature may define the broad range of suitable habitats, whereas relative humidity, grape phenology, precipitation, and sunshine duration may further modify population density within this range. This multi-factor explanation helps account for the observed spatial heterogeneity of *A. lucorum* even under similar regional climatic conditions. Therefore, population occurrence in this region is better understood as the result of the combined effects of multiple environmental and host-related factors rather than the influence of any single driver.

### 4.3. Study Limitations and Future Directions

Several limitations should be acknowledged. First, this study analyzed the seasonal dynamics of *A. lucorum* mainly at the adult trap-catch level, but did not systematically examine overwintering eggs, nymphal populations, or generational structure. Therefore, it remains difficult to determine whether the observed population peaks were caused by single generations, overlapping cohorts, or immigration from surrounding habitats. Second, the population density used in this study was mainly based on adult pheromone-trap catches and therefore represents adult trap-based occurrence rather than total population pressure. Because nymphs are not captured by pheromone traps but can also damage grape tissues, the absence of nymphal data may affect the assessment of actual damage risk and control timing. In addition, locally validated harmfulness or economic thresholds for *A. lucorum* in wine grapes are still lacking. Thus, the model outputs should be interpreted as early-warning indicators of adult occurrence risk rather than direct criteria for pesticide application. Third, understory vegetation, field weeds, natural enemies, interspecific competition, and vineyard management practices, such as pruning, fertilization, irrigation, and pesticide application, were not included in the analysis. These factors may influence local population dynamics and partly explain the unexplained variation in the model. Fourth, the five landscape units are unreplicated spatial blocks rather than replicated treatment levels, and the sampling points within each unit are spatially clustered subsamples. Consequently, among-unit differences are confounded with geographic location, elevation, and climate, and variable importance such as the high SHAP ranking of elevation should be interpreted as confounded associations rather than independent causal effects.

Future studies should integrate adult trap catches, nymphal surveys, plant injury assessment, grape phenology, and locally validated economic thresholds to better support pest-management decisions. In addition, multi-year monitoring, vineyard management information, biotic factors, remote-sensing data, and landscape connectivity should be incorporated to improve the reliability and practical applicability of pest forecasting models. Where feasible, a replicated or spatially interspersed sampling design would also help disentangle landscape-unit effects from confounded environmental gradients.

## 5. Conclusions

This study characterized the seasonal and spatial variation in adult *A. lucorum* trap-based density in an arid wine-grape region during 2024–2025. Adult catches were low in May, increased from June to July, peaked during August–September, and remained relatively high in October, with higher overall density in 2025 than in 2024. LOWESS smoothing identified a general three-stage seasonal pattern of rapid growth, peak occurrence, and decline. Spatial analyses showed that adult trap catches were aggregated in most landscape unit–phenological phase combinations, although hotspot patterns varied among years, landscape units, and phenological stages. The XGBoost-SHAP model showed good predictive performance and identified grape phenology, elevation, relative humidity, sunshine duration, and temperature as the main predictors of adult trap-based density. Partial dependence analysis suggested that predicted adult density increased during later grape phenological stages and under higher relative humidity. Overall, combining pheromone-trap monitoring with phenological and meteorological information can support early warning of adult *A. lucorum* occurrence. However, the model outputs represent adult trap-based risk and should not be used directly as pesticide-application criteria. Practical pest-management decisions should also include nymphal surveys, plant injury assessment, grape phenology, and locally validated economic thresholds.

## Figures and Tables

**Table 1:** Variance inflation factor (VIF) values for meteorological, phenological, and topographic predictors used in the XGBoost model.

Predictor Category	Feature	VIF
Topographic	Elevation	1.226979
	Slope	1.207501
	Aspect	1.411563
	Curvature	1.09529
	Shade Index	1.363094
Meteorological	Temperature (°C)	1.547138
	Relative Humidity	3.434466
	Wind Speed (m/s)	1.265065
	Sunshine Duration (h)	1.426231
	Precipitation (mm)	1.158287
Phenological	Grape Phenological DOY	3.955939

## Data Availability

Raw data supporting the conclusions of this article are available from the corresponding author on reasonable request.

## Supplementary Materials

The following supporting information can be downloaded at: https://www.mdpi.com/article/10.3390/insects17070719/s1, The Supplementary Materials of this study comprise eight supplementary tables (Tables S1–S8). Table S1. Phenological stages and core characteristic indicators of Cabernet Sauvignon in the eastern foothills of Helan Mountain, Ningxia. Table S2. Phenological Calendar, Date Range, Duration and DOY Range of Cabernet Sauvignon Wine Grapes in the Eastern Foothills of Helan Mountain, Ningxia, China. Table S3. Parameter combinations used for LOWESS sensitivity analysis. Table S4. Summary of sensitivity analysis for phase boundary estimates. Table S5. Optimal variogram models and fitted parameters for ordinary kriging interpolation across years, landscape units, and phenological phases. Table S6. Leave-one-out cross-validation performance of ordinary kriging models across years, landscape units, and phenological phases. Table S7. Spatial autocorrelation and hotspot-related statistics of adult *A. lucorum* trap density across years, landscape units, and phenological phases. Table S8. Top 20 feature pairs with the highest mean absolute SHAP interaction values for adult *A. lucorum* trap-based density prediction.
