# Supplementary material for: Spatiotemporal Dynamics and Outbreak Risk of Apolygus lucorum in Semi-Arid Wine Grape Regions: An Analysis Based on Multi-Factor Drivers and Machine Learning Models
Source: Insects. 2026 Jul 11;17(7):719. doi: 10.3390/insects17070719 (PMC13411125; doi:10.3390/insects17070719)
Supplement: Supplementary file 1 [file insects-17-00719-s001.zip › insects-4369798-supplementary.pdf]

insects

MDPI

Article

Spatiotemporal dynamics and outbreak risk of *Apolygus lucorum* in semi-arid wine grape regions: An analysis based on multi-factor drivers and machine learning models

Supplementary Materials

Table S1. Phenological stages and core characteristic indicators of Cabernet Sauvignon in the eastern foothills of Helan Mountain, Ningxia

Phenological Stage	Core Characteristic Indicators for Cabernet Sauvignon
Bleeding Stage	After soil thawing in spring, root activity resumes, a large amount of sap flows out from branch pruning cuts, buds have not yet sprouted, and the tree enters the pre-preparation stage of physiological activity
Bud Break Stage	5% of bud scales crack, the villous layer breaks, young leaves are exposed and appear light pink, and new shoots begin to elongate and grow
Shoot Growth Stage	New shoots elongate rapidly, leaves fully expand, inflorescences complete differentiation, young leaves continue to grow, and vegetative growth enters a vigorous period
Flowering and Fruit Set Stage	Start of flowering: 5% of caps fall off; Full bloom: 50% of caps fall off; Fruit set stage: entered after 95% of flowers complete flower drop
Berry Swelling Stage	Berries expand rapidly, green young fruits continue to grow, seed embryos complete development, fruit acidity accumulates rapidly, and nutrients are reserved for the veraison stage
Veraison Stage	5% of berries begin to change color, from green to iconic blue-purple, berries gradually soften, sugar rises rapidly, and acidity continues to decrease
Berry Ripening Stage	Berries fully show the inherent blue-black color of Cabernet Sauvignon, accumulation of sugar, phenolic substances and aromatic substances is completed, and acidity drops to the suitable harvest level
Shoot Lignification Stage	New shoots begin to lignify from the base, epidermis changes from green to brown, winter buds are fully developed, and leaf nutrients continuously flow back to branches and roots
Leaf Fall and Dormancy Stage	Leaves fall naturally, winter buds enter deep dormancy, tree life activity drops to the lowest, and physiological dormancy preparation before overwintering is completed

Table S2. Phenological Calendar, Date Range, Duration and DOY Range of Cabernet Sauvignon Wine Grapes in the Eastern Foothills of Helan Mountain, Ningxia, China

Phenological Stage	Corresponding Date Range	Duration (Days)	DOY Range
Bleeding Stage	Late March - Early April	10-15	80-105
Bud Break Stage	Mid-late April - Early May	10-15	110-125
Shoot Growth Stage	Early May - Early June	30-35	120-155
Flowering and Fruit Set Stage	Early June - Mid June	15-20	155-170
Berry Swelling Stage	Mid June - Late July	40-45	165-210
Veraison Stage	Late July - Mid August	20-30	205-235
Berry Ripening Stage	Late September - Early October	40-50	230-285
Shoot Lignification Stage	Mid September - Late October	30-40	255-295
Leaf Fall and Dormancy Stage	Late October - Late March of the following year	150-160	290 - 85 of the following year

Table S3. Parameter combinations used for LOWESS sensitivity analysis.

frac	low_thresh	high_thresh
0.25	0.1	0.9
0.25	0.2	0.8
0.25	0.25	0.75
0.3	0.1	0.9
0.3	0.2	0.8
0.3	0.25	0.75
0.4	0.1	0.9
0.4	0.2	0.8
0.4	0.25	0.75
0.5	0.1	0.9
0.5	0.2	0.8
0.5	0.25	0.75

Table S4. Summary of sensitivity analysis for phase boundary estimates.

Year	2024					2025				
Landscape Unit	LU1	LU2	LU3	LU4	LU5	LU1	LU2	LU3	LU4	LU5
Start_min	3.819	1.683	2.196	1.000	3.819	1.000	1.171	2.281	1.000	1.171
Start_max	7.236	5.442	5.528	4.246	7.492	2.452	5.955	8.432	1.171	4.246
Start_range	3.417	3.759	3.332	3.246	3.673	1.452	4.784	6.151	0.171	3.075
Start_std	1.196	1.406	1.230	1.211	1.202	0.506	1.580	2.254	0.053	1.239
Peak_min	12.020	10.055	10.055	7.065	12.020	16.035	12.960	12.960	5.015	12.960
Peak_max	13.985	11.935	12.960	9.030	13.985	18.000	13.985	13.985	7.920	13.985
Peak_range	1.965	1.879	2.905	1.965	1.965	1.965	1.025	1.025	2.905	1.025
Peak_std	0.818	0.779	1.049	0.962	0.818	0.818	0.444	0.444	1.080	0.492
Decline_min	12.704	12.447	12.618	7.920	13.729	16.804	13.558	13.558	7.663	13.387
Decline_max	15.608	13.899	14.241	11.080	15.864	18.000	16.206	15.608	10.055	15.181
Decline_range	2.905	1.452	1.623	3.161	2.136	1.196	2.648	2.050	2.392	1.794
Decline_std	0.850	0.432	0.487	1.007	0.670	0.331	0.855	0.620	0.666	0.578

Table S5. Optimal variogram models and fitted parameters for ordinary kriging interpolation across years, landscape units, and phenological phases.

Year	LU	Phase	Best_Model	Nugget	Partial_Sill	Range	Total_Sill	WRSS
2024	LU1	Phase I	spherical	2.70	13.05	0.01	15.75	141.33
		Phase II	gaussian	231.48	1852.54	0.01	2084.02	201409.69
		Phase III	spherical	239.41	785.04	0.01	1024.45	333634.36
	LU2	Phase I	gaussian	3.86	34.92	0.011531	38.77	14.38
		Phase II	gaussian	66.26	645.31	0.01	711.58	25086.07
		Phase III	gaussian	47.75	473.49	0.01	521.25	11105.26
	LU3	Phase I	spherical	0.48	42.91	0.01	43.39	441.25
		Phase II	spherical	0.00	1294.16	0.01	1294.16	187439.47
		Phase III	spherical	0.00	1341.39	0.010001	1341.39	255391.39
	LU4	Phase I	spherical	2.33	0.27	0.01	2.60	4.02
		Phase II	exponential	21.22	196.25	0.018898	217.47	2519.10
		Phase III	gaussian	19.70	136.91	0.01872	156.61	559.39
	LU5	Phase I	exponential	2.62	21.69	0.01	24.31	30.68
		Phase II	exponential	20.26	365.69	0.01	385.95	8789.25
		Phase III	exponential	12.84	36.67	0.01	49.52	679.97
2025	LU1	Phase I	exponential	0.09	0.43	0.01	0.52	0.08
		Phase II	exponential	36.88	74.33	0.01	111.21	13671.84
		Phase III	exponential	20.77	44.49	0.01	65.26	4370.41
	LU2	Phase I	spherical	0.10	0.38	0.01	0.48	0.11
		Phase II	spherical	0.00	5.45	0.014798	5.45	1.20
		Phase III	spherical	0.00	6.90	0.014798	6.90	18.19
	LU3	Phase I	exponential	0.81	3.09	0.01	3.90	7.24
		Phase II	spherical	0.05	0.94	0.01	0.99	0.14
		Phase III	spherical	0.00	20.94	0.01	20.94	72.25
	LU4	Phase I	gaussian	0.00	0.09	0.01	0.10	0.00
		Phase II	spherical	0.35	0.11	0.01	0.46	0.13
		Phase III	gaussian	0.00	0.07	0.01	0.07	0.00
	LU5	Phase I	exponential	0.22	1.59	0.01	1.81	1.32
		Phase II	spherical	0.00	8.42	0.010049	8.42	6.15
		Phase III	exponential	3.76	20.31	0.01	24.07	245.65

Table S6. Leave-one-out cross-validation performance of ordinary kriging models across years, landscape units, and phenological phases.

Year	LU	Phase	ME	MAE	RMSE	RMSSE	R^2
2024	LU1	Phase I	0.007219	1.236862	1.524929	0.580577	0.66293
		Phase II	0.025085	6.965358	8.363474	0.434165	0.811501
		Phase III	-0.048559	7.492374	9.46783	0.444168	0.802714
	LU2	Phase I	0.036633	2.107962	2.355151	0.891225	0.205718
		Phase II	0.041245	4.821425	5.93844	0.522724	0.726759
		Phase III	0.073542	4.058849	4.93683	0.507313	0.742634
	LU3	Phase I	0.079457	1.929146	2.349749	0.688461	0.526022
		Phase II	-0.142694	3.506519	4.628997	0.287725	0.917214
		Phase III	0.158716	4.573138	5.767541	0.335824	0.887222
	LU4	Phase I	-0.03347	2.200469	2.670071	1.696637	-1.878579
		Phase II	0.020228	3.436691	4.480977	0.590443	0.651377
		Phase III	0.037979	2.996757	3.490956	0.667129	0.554939
	LU5	Phase I	-0.035284	1.593775	1.979627	0.812707	0.339507
		Phase II	0.021431	3.858806	4.797339	0.521916	0.727604
		Phase III	0.001453	2.58141	3.06966	0.686473	0.528755
2025	LU1	Phase I	0.006845	0.168824	0.260053	0.567798	0.677606
		Phase II	0.100158	4.000753	5.532322	0.697406	0.513625
		Phase III	0.079217	2.934621	4.09269	0.68163	0.53538
	LU2	Phase I	0.002756	0.192318	0.237778	0.440937	0.805574
		Phase II	-0.048584	0.279093	0.371696	0.290503	0.915608
		Phase III	0.001005	0.078805	0.133394	0.11126	0.987621
	LU3	Phase I	-0.012738	0.457637	0.644569	0.542704	0.705472
		Phase II	-0.009766	0.100571	0.166464	0.309421	0.904259
		Phase III	-0.001831	0.163487	0.313288	0.1531	0.97656
	LU4	Phase I	-0.002878	0.016292	0.02491	0.168213	0.971704
		Phase II	0.111109	1.088553	1.328541	2.065752	-3.26733
		Phase III	0.002495	0.02289	0.027898	0.230877	0.946696
	LU5	Phase I	-0.003637	0.309939	0.506148	0.687473	0.527381
		Phase II	-0.027446	0.326536	0.521294	0.345873	0.880372
		Phase III	-0.001318	1.075072	1.653971	0.579281	0.664434

Table S7. Spatial autocorrelation and hotspot-related statistics of adult *A. lucorum* trap density across years, landscape units, and phenological phases.

Year	LU	Phase	Moran_I	Moran_*P*	Gi_mean	Gi_std	Hot_ratio
2024	LU1	Phase I	0.275019	***	0.208366	1.992681	0.266667
		Phase II	0.308037	***	0.21527	2.091986	0.266667
		Phase III	0.30836	***	0.214934	2.092784	0.266667
	LU2	Phase I	0.12813	**	-0.243983	1.827703	0.066667
		Phase II	0.291194	***	-0.112508	2.203955	0.3
		Phase III	0.296307	***	-0.137047	2.239398	0.3
	LU3	Phase I	0.298932	***	0.116596	1.925851	0.2
		Phase II	0.432436	***	0.094546	2.258651	0.266667
		Phase III	0.425736	***	0.08807	2.250278	0.266667
	LU4	Phase I	0.05993	0.180	-0.194762	1.170399	0.033333
		Phase II	0.150169	**	-0.103932	1.436257	0.033333
		Phase III	0.138174	*	-0.186439	1.382583	0.033333
	LU5	Phase I	0.158707	**	0.24769	1.707901	0.166667
		Phase II	0.226214	***	0.207841	1.787256	0.066667
		Phase III	0.184115	**	0.170791	1.714939	0.1
2025	LU1	Phase I	0.124919	*	-0.029583	1.292034	0.166667
		Phase II	0.141709	*	-0.118056	1.373258	0.133333
		Phase III	0.141971	*	-0.115719	1.376116	0.133333
	LU2	Phase I	0.174772	**	0.072095	1.644519	0.2
		Phase II	0.25077	***	0.507659	2.015004	0.4
		Phase III	0.349616	***	0.089145	2.253951	0.3
	LU3	Phase I	0.10322	*	0.230655	1.059811	0
		Phase II	0.201294	***	0.253377	1.732481	0.133333
		Phase III	0.421008	***	0.086394	2.182181	0.3
	LU4	Phase I	0.320142	***	0.285267	2.14237	0.266667
		Phase II	0.040906	0.284	-0.586577	1.130114	0
		Phase III	0.331886	***	-0.14553	2.09066	0.1
	LU5	Phase I	0.062092	0.167	0.130455	1.177989	0
		Phase II	0.218698	***	0.342127	1.829776	0.233333
		Phase III	0.146926	**	0.131387	1.606411	0.2

* $P < 0.05$; **$P < 0.01$; ***$P < 0.001$

Table S8. Top 20 feature pairs with the highest mean absolute SHAP interaction values for adult *A. lucorum* trap-based density prediction.

Feature_Pair	Mean_Abs_SHAP_Interaction
Elevation × Grape Phenology (DOY)	1.6612085
Elevation × Relative humidity (%)	1.0538572
Temperature (°C) × Grape Phenology (DOY)	0.7658407
Elevation × Temperature (°C)	0.735726
Sunshine duration (h) × Grape Phenology (DOY)	0.6667807
Temperature (°C) × Relative humidity (%)	0.660854
Relative humidity (%) × Sunshine duration (h)	0.6037015
Relative humidity (%) × Grape Phenology (DOY)	0.51249266
Shading Index × Grape Phenology (DOY)	0.50155294
Elevation × Wind speed (m/s)	0.4633701
Temperature (°C) × Sunshine duration (h)	0.37971058
Relative humidity (%) × Precipitation (mm)	0.33268797
Precipitation (mm) × Grape Phenology (DOY)	0.30878577
Elevation × Sunshine duration (h)	0.298495
Wind speed (m/s) × Precipitation (mm)	0.28840724
